# *Rhodiola rosea* L. roots powder strongly reduces anxiety and corticosterone level induced by chronic stress in a murine model

**DOI:** 10.1186/s40780-025-00532-4

**Published:** 2026-01-20

**Authors:** Camille Lelong, Laurence Ris, Oksana Sytar, Sylvie Defrère, Agnès Villers

**Affiliations:** 1 Department of Neurosciences, Research Institute for Health Science and Technology, University of Mons, Avenue du Champ de Mars 8, Mons, 7000 Belgium; 2 Botalys – Research & Development Department, Avenue des artisans 8 box 6, Ghislenghien, 7822 Belgium; 3 Phytopathology, Microbial and Molecular Farming Lab, Haute Ecole Provinciale du Hainaut – Condorcet, Rue Paul Pastur 11, Ath, 7800 Belgium

**Keywords:** *Rhodiola rosea L*, Chronic stress, Murine model, Salidroside, Cortisol, Mental health

## Abstract

Chronic stress disrupts physiological and psychological homeostasis, yet effective therapeutic strategies remain limited. This study investigated the adaptogenic effects of Rhodiola rosea root powder (standardized to 3% salidroside) on stress-induced behavioral and physiological changes in a murine model of chronic mild stress. Female C57BL/6 mice were exposed to a 19-day chronic mild stress protocol and received daily oral supplementation of Rhodiola rosea root powder, administered in gummies to ensure accurate and stress-free intake, or a placebo. At the end of the experiment, behavioral outcomes were assessed using the Elevated Plus Maze (EPM) and Open Field (OF) tests. Compared with stressed controls, stressed treated mice demonstrated significant improvements. In the EPM, treated mice showed significantly higher locomotor activity (greater distance and speed), with more open-arm entries and time, as well as increased head dips, indicating reduced anxiety and enhanced exploration. In the OF test, they also displayed greater locomotion and more center-zone entries, both reaching statistical significance and supporting reduced anxiety-like behavior. These behavioral improvements were accompanied by a significant reduction in serum corticosterone levels, indicating modulation of the physiological stress response. Together, the findings support the anxiolytic and adaptogenic properties of Rhodiola rosea root powder and highlight its potential as a natural intervention for managing chronic stress. Future studies should investigate the long-term efficacy and mechanisms of its bioactive compounds in stress resilience and mental health.

**Graphical Abstract:**

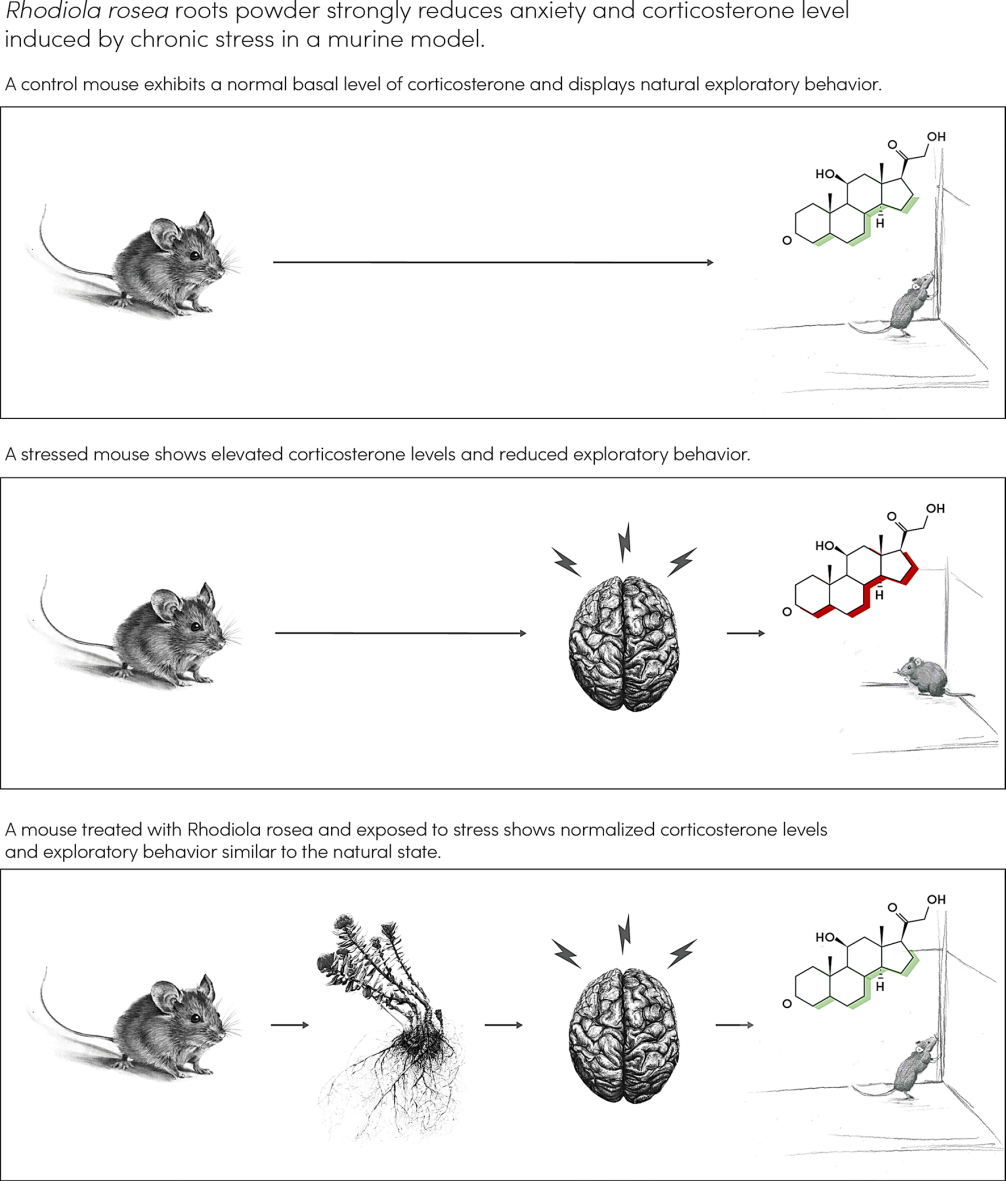

## Introduction

A certain amount of stress can sometimes be beneficial, providing the drive and energy needed to handle situations like exams or work deadlines [1]. However, chronic and excessive stress can lead to cumulative negative effects on health, through a phenomenon described by the concept of “allostatic load” [1–4]. This concept refers to a shifted or altered state of homeostasis resulting from prolonged, excessive, or poorly regulated allostatic responses [1, 5, 6]. Moreover, extensive research demonstrates that prolonged exposure to chronic stress is linked to adverse effects, disrupting the functioning of the immune, cardiovascular, neuroendocrine, and central nervous systems [4, 7, 8].

In the present study, a murine model of chronic stress was established through repeated exposure to mild stressors. This approach is well-documented in the literature as a reliable paradigm to induce both physiological and behavioral alterations that resemble stress-related disorders in humans. Previous work has demonstrated that chronic early-life stress in mice leads to acute and long-lasting neuroendocrine and cognitive abnormalities (Courtney J. [9, 10]). Moreover, rodent models of chronic stress have been widely validated for their ability to induce measurable alterations in exploratory behavior, anxiety-like responses, and hypothalamic–pituitary–adrenal (HPA) axis activity [11, 12]. Chronic behavioral stress paradigms in mice can generally be classified into models emphasizing predominantly social stressors or those employing non-social aversive stimuli, with some protocols incorporating a combination of both [13]. Rodent models combining physical and psychological stressors have yielded key insights into stress physiology [14]. Such models provide a robust framework for evaluating the efficacy of potential adaptogenic or anxiolytic interventions [15, 16] in mitigating the behavioral and physiological consequences of sustained stress exposure.

Despite the considerable negative impact of chronic stress, current pharmacological treatments show a significant gap [17]. Many vitamin supplements, and prescription medications primarily target individual symptoms rather than addressing stress in a more holistic manner. Furthermore, psychiatric drugs such as antidepressants, anxiolytics, or beta-blockers are generally prescribed for more severe conditions like depression or anxiety. Their use carries risks of overtreatment, including serious side effects and potential dependency [18].

Medicinal plants have historically played a significant role in drug discovery, offering a wide array of bioactive compounds [19–21]. However, the development of innovative technologies for obtaining pure, standardized, and sustainably cultivated botanicals with high levels of specific secondary metabolites is essential to produce plant-derived natural products.

*Rhodiola rosea L.* is gaining significant attention among medicinal plants for its potential to alleviate stress. Recognized as an “adaptogen”, it is a substance that enhances the body’s resistance to stress without disrupting normal biological functions while promoting physiological balance [22]. A plant is considered adaptogenic when it helps the body regain balance and adapt to various types of stress. Adaptogens act as mild stress mimetics at low doses, stimulating adaptive stress-response pathways and supporting neuroendocrine and immune functions, which explains their traditional use against fatigue, stress, and aging [23]. To be classified as an adaptogen, it must meet three specific criteria: it increases the body’s resistance, maintains or restores physiological balance, and is non-toxic. Its therapeutic effects are attributed, among others, to active secondary metabolites that reduce cortisol levels [24]. Supported by its long history in traditional medicine and extensive scientific research [25–27], the European Medicines Agency (EMA) issued a herbal monograph, approving *Rhodiola rosea* L. rhizoma et radix for traditional use as an adaptogen to temporarily relieve stress-related symptoms, including fatigue, exhaustion, and general weakness [18, 28, 29]. In addition to its recognition by the EMA, *Rhodiola rosea* L. is officially listed in the United States Pharmacopeia and is included in the pharmacopoeias of several countries in the Eurasian Economic Union, such as Russia and Belarus, where it is used in officinal medicine. These listings reflect a growing international consensus on the relevance of its therapeutic potential and support its integration into both traditional and modern medical frameworks.

Salidroside and rosavins, the primary bioactive compounds in *Rhodiola rosea* L., modulate the hypothalamic–pituitary–adrenal (HPA) axis, although their precise mechanisms of action remain only partially understood [30]. The adaptogenic effects are mainly attributed to salidroside. Centrally, one study demonstrated that salidroside reduces c-Fos expression in the paraventricular nucleus (PVN) of the hypothalamus, a neuronal activation marker associated with corticotropin-releasing hormone (CRH) secretion. This inhibition of hypothalamic activity leads to a decrease in CRH release, thereby limiting the initial activation of the HPA axis [31]. Yang et al. [32] further showed that salidroside modulates HPA axis activity by downregulating hypothalamic CRH expression and reducing serum corticosterone levels in olfactory bulbectomized rats, suggesting an antidepressant effect partially mediated by HPA regulation. However, current data remain insufficient to establish whether *Rhodiola rosea* L. significantly influences ACTH or cortisol release.

More than 140 compounds have been isolated from *Rhodiola rosea L.* [29, 33]. Among these, salidroside (rhodioloside), trans-cinnamyl alcohol glycoside compounds (such as rosin, rosavin and rosarin), and tyrosol are considered the most critical constituents for its therapeutic activity [34–36]. Notably, rosavin is unique to *Rhodiola rosea L.* within the *Rhodiola* genus, whereas salidroside and tyrosol are commonly found in other *Rhodiola* species [37, 38].

Typically, preparations of Rhodiola rosea L. are standardized to contain 1% salidroside and 3% rosavin [39–41]. Salidroside and rosavin are generally regarded as the key adaptogenic compounds in herbal medicinal products and dietary supplements. Several preclinical [31, 42–47] and clinical studies [48–52] have demonstrated that *Rhodiola rosea* root extracts may serve as effective natural remedies for improving mental and cognitive performance under stress. However, these studies have exclusively focused on root extracts, while no published research has yet examined the effects of the whole root powder.

Root powder preserves the complete phytochemical spectrum of the plant [53], including minor compounds that may act synergistically rather than isolating individual molecules [33, 54]. The powder used in this study was standardized to 3% salidroside, higher than typical extract formulations (1% salidroside, 3% rosavin), making it a unique preparation that could elicit different or stronger adaptogenic effects.

To the best of our knowledge, this is the first experimental research to describe the anxiolytic and corticosterone-reducing effects of whole root powder standardized to 3% salidroside in a chronic stress model. Unlike prior work that often employed acute stress paradigms or tested extracts in healthy animals, our investigation specifically evaluated root powder under chronic mild stress conditions, a model more relevant to human stress-related disorders. Furthermore, administration in a gummy format ensured accurate, stress-free dosing and represents a practical delivery system translatable to human use. Together, these findings highlight that *Rhodiola rosea* root powder offers a minimally processed, sustainable, and effective alternative to standardized extracts, expanding the therapeutic potential of this adaptogenic plant for stress management.

The aim of this study was to evaluate the potential effects of *Rhodiola rosea* L. root powder, with high level of salidroside (3%), on a murine model of chronic stress. For this purpose, a murine model of chronic stress was established using repeated mild stress exposure. The impact of daily *Rhodiola rosea L.* root powder administration during the stress period was then assessed. At the end of the experiment, stress levels were evaluated by measuring anxiety-like behavior and corticosterone levels. The results confirm that *Rhodiola rosea* L. root powder significantly modulates both physiological and behavioral markers of stress.

## Material & methods

### Animals

The guidelines for animal welfare were approved by the Committee on Animal Research of the Université de Mons (ref RI-01501).

8-weeks-old C57BL6 female mice were supplied by Charles River (agreement: C 69 208 1301). Mice were acclimated for 1 week in the animal house at the University of Mons (agreement: LA1500550T) and were sustained in a 12-hour light–dark cycle. The animals were housed in groups (6 mice per cage) and kept in a room with controlled temperature and humidity, with food and water available ad libitum. At the end of the experiment, mice were anesthetized by isoflurane inhalation and euthanized by decapitation for blood collection. Blood samples were collected two hours after the final behavioral test.

To avoid stress-related bias due to fights, which are often observed in cohorts of male mice, only female mice were used for this study.

### Botanical compound and measurement of salidroside, rosin, rosarin and rosavin (UHPLC)

The *Rhodiola rosea L.* roots powder used in this study (batch number RR_2405_001) was produced by Botalys (Ghislenghien, Belgium). A carefully selected cultivar of *Rhodiola rosea* L. is hydroponically cultivated in an innovative vertical farming technology, with a strict control of growing conditions (BOTALYS is FSSC22000 certified), allowing a reproducible chemical composition of the roots from one batch to another, and containing high content of active compounds. At the end of the culture the fresh roots are harvested and dried. The dried *Rhodiola rosea L.* roots is then grounded to obtain powder. The powder is sieved on 300 µm. The final product is analysed for salidroside and Rosavins content before release.

The identity of the *Rhodiola rosea* L. roots was verified by DNA sequencing. The sequence of the DNA fragment obtained from Botalys *Rhodiola rosea* L. root powder presents 99.66% of similarity with *Rhodiola rosea L.* sequence recorded in the Genbank genetic database. Moreover, the active compounds of *Rhodiola rosea L.*, i.e. salidroside and rosavins are detected in the Botalys *Rhodiola rosea L.*.

Compounds were extracted and analyzed as follows: the dry powder of *Rhodiola rosea L.* (0.1 g) was extracted in 10 mL of 70% methanol during 45 minutes in an ultrasonic bath. After extraction, the solution was filtered through a 0.22-μm Millipore filter and used for UHPLC analysis. The content of salidroside, rosin, rosarin and rosavin was quantified using a SHIMADZU UHPLC LC-20 ADXR modular system, which included an SPD-40 V detector, SIL-40C autosampler, LC-40B XR pump, CTO-40C column oven, and a Shim-pack GIST C18 2 μm column (150 ×2.1 mm). A 2 μL sample injection volume was used, with analysis conducted at 40 °C and detection at 192 nm. Separation was achieved using a linear gradient elution with solvent A (0.1% phosphoric acid solution) and solvent B (acetonitrile). The gradient was as follows: *t* = 0 min, 98% A; *t* = 13.33 min, 88% A; *t* = 22 min, 30% A; and *t* = 22.66 min, 98% A. The flow rate was set to 0.45 mL/min. Calibration curves were established using standards of salidroside, rosin, rosarin and rosavin purchased from Sigma-Aldrich Merck.

The concentration was measured using the following formula:

$${\rm{Percentage\;of\;molecule}} = {{ppm mesured x extraction volume} \over {mass x 10 000}}$$

The total rosavins content is calculated as the sum of the percentages of rosin, rosavin, and rosarin.

### Treatment

To avoid the stress associated with gavage, the daily treatment was orally administrated to the mice in the form of a gummies ensuring both precise and controlled intake.

The gummies were prepared as follow: 100 ml of water and 60 g of granulated sugar were mixed and brought to a boil for a few minutes. Then, 3 sheets of gelatin (or 6 g of powdered gelatin) and 4 ml of raspberry flavoring were added to the mixture. The solution was left to cool to ±70 °C. For “Rhodiola gummies”, 67.2 mg/mL of *Rhodiola rosea* L. root powder was then incorporated to the solution and homogenized. For “placebo gummies”, nothing was added to the mixture. Next, 1 ml of the solution was poured into each cavity of a silicone mold which was placed in the fridge until gummies solidification. Finally, the gummies were cut into four equal and standardized portions, ensuring that each animal received 16.8 mg of *Rhodiola rosea* L. root powder per dose.

The selected dose of 800 mg/kg/day was determined based on previous research findings [43, 44, 55]. Additionally, a prior toxicity study (unpublished data) confirmed the safety of the product at a dose of 2000 mg/kg/day.

Each mouse received one gummy per day, always at the same time, and administration was performed individually in a separate cage to ensure full ingestion. Cages were visually inspected to confirm that each mouse consumed the entire portion without fragmentation or leftovers. Animals were observed for approximately 10 minutes after administration to verify complete consumption. Behavioral testing was conducted approximately one hour after gummy administration. Stressful events followed one another without interruption, in accordance with the protocol described.

### Induction of chronical stress

To induce mild stress in the experimental group, a sequence of stressors was applied following a standardized protocol. These stressors were selected to mimic environmental and physiological challenges, ensuring a controlled yet multifaceted stress exposure [56–58]. The protocol consisted of the following sequential stress-inducing conditions:

- Cage tilting: the home cage was inclined at a 30° angle for a duration of 6 hours to disrupt spatial stability.
- Olfactory stress: subjects were exposed to the odor of lemon essential oil for 24 hours, a stimulus known to induce mild discomfort in rodents.
- Food and water deprivation: access to food and water was restricted for a period of 18 hours to simulate transient resource scarcity.
- Bedding reduction: the quantity of bedding material was significantly reduced for 6 hours, limiting comfort and thermoregulation.
- Continuous light exposure: a 24-hour period of uninterrupted light exposure was implemented to disrupt circadian rhythms.
- Social isolation: subjects were housed individually for a total of 3 days to induce psychosocial stress.
- Physical restraint: finally, animals were subjected to a 30-minute physical restraint session to elicit an acute stress response.

This multi-component protocol was designed to elicit a cumulative stress response, modeling a mild but persistent stress condition.

### Elevated plus maze (EPM) test

The EPM is a widely used tool in behavioral research to assess stress and anxiety in rodents, particularly mice [59]. This apparatus consists of two open arms and two closed arms arranged in a cross shape, elevated above the ground. The test leverages the natural conflict in mice between their exploratory instincts and their innate aversion to open, elevated spaces. By observing the time spent in the open arms versus the closed arms, researchers can quantify the mouse’s anxiety levels. For instance, a more stressed mouse will spend more time in the closed arms, which are perceived as safer. The animals were monitored for a duration of 5 minutes using the EthoVision tracking system. Behavioral data were recorded and subsequently analyzed following the statistical methods outlined below. The dimensions of the EPM were as follows: the open arms measured 35 cm each, the closed arms were 35 cm each, the corridor width was 5 cm, the walls of the closed arms were 20 cm in height and the apparatus was elevated 60 cm above the floor (Ugo Basile).

In this study, the Elevated Plus Maze (together with the Open Field test) was employed across all three experimental phases with clearly defined time points. In Phase I, mice (both control and stressed groups) were tested at three periods: baseline (before any stress exposure), pre-stress (D + 19), and post-stress (D + 26) to assess the effects of the chronic mild stress protocol. In Phase II, the same tests were used at two time points (D0 and D14) without any treatment or stress to evaluate possible habituation effects. In Phase III, a finalized protocol compared stressed mice receiving daily Rhodiola rosea L. root powder (D + 15 to D + 33) with stressed but untreated controls, with a single behavioral assessment performed at the end of the stress period (D + 33) before sacrifice and blood collection.

### Openfield (OF) test

The OF test is a common method for assessing stress and anxiety in mice [59]. It involves placing the animal in a large open arena and observing its movements. Anxious mice tend to stay near the walls, while less anxious ones explore the center. Key measures include distance traveled, time spent in the center, and exploratory behavior, providing insights into emotional state and treatment effects. Animals were monitored for a duration of 5 minutes using the EthoVision tracking system. Behavioral data were collected and analyzed using the statistical methods detailed below. The dimensions of the experimental arena were 40 × 40 × 40 cm (Ugo Basile). The wall was 40 cm in height.

### Corticosterone measurement

Corticosterone levels were measured using the ELISA kit from Enzo Life Sciences (ADI-901–097). During the euthanasia of the animals, blood was collected and kept at 4 °C for 24 hours. The blood was then centrifuged, and the supernatant was carefully collected. The supernatant was stored at −80 °C until further analysis. The dosing was performed according to the kit’s recommendations.

### Statistical analysis

All values are expressed as the mean ± standard error of the mean (SEM). Graphs and statistical analyses were performed using GraphPad Prism version 10.

For the first phase of result, after verifying the normality assumption, a two-way ANOVA for repeated measures was conducted, followed by Fisher’s post hoc test for multiple comparisons. Corticosterone levels, which is not a repeated measure, and which did not meet normality assumptions, were analyzed using a non-parametric Mann-Whitney test.

For the second phase of result, paired t-tests were used for data that followed a normal distribution, whereas Wilcoxon signed-rank tests were applied for non-normally distributed results.

For the last phase of result, unpaired t-tests were performed for normally distributed data, while Mann-Whitney tests were used for non-parametric comparisons. A p-value of less than 0.05 was considered statistically significant.

## Results

### Salidroside and Rosavins level in Rhodiola rosea L. roots powder

**Fig. 1:**
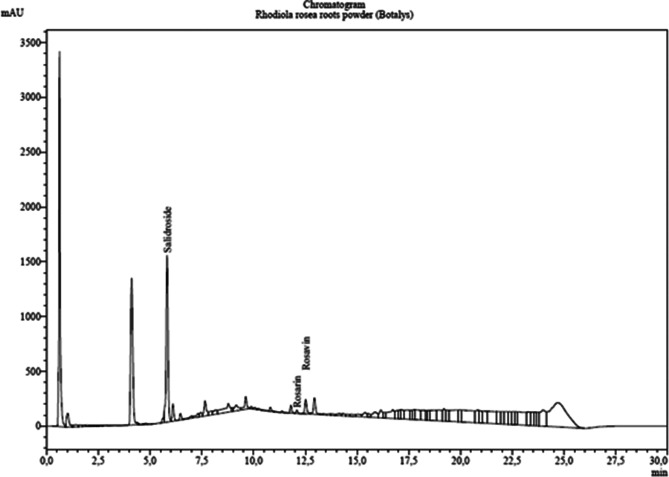
Chromatogram of *Rhodiola rosea L*. root powder (Botalys)

An HPLC method was developed for the identification of five marker compounds of *Rhodiola rosea* (salidroside, rosarin, rosavin, rosin, and rosiridin). A similar analytical objective had previously been reported by [60, 61]. The preliminary quality assessment did not reveal the presence of rosavin or rosiridin in our sample (Fig. 1, Table 1). Salidroside and rosavins (rosin, rosavin, rosarin) were measured by UHPLC in the *Rhodiola rosea L.* roots powder used in this study. A content of 3.0% (g/100 g of dry matter) salidroside and 0.8% rosavins (rosin, rosavin, rosarin) were measured (Table 1).

**Table 1:** Analysis results of Rhodiola rosea L. root powder (*n* = 3; Botalys)

Name	Ret. Time	Area	Height	Conc.	Unit
Salidroside	5.84 ± 0.04	9297057 ± 34892	1522896 ± 10520	277.67 ± 1.04	ppm
Rosarin	12.11 ± 0.04	201836 ± 13223	31366 ± 834	12.22 ± 0.80	ppm
Rosavin	-	-	-	-	ppm
Rosiridin	-	-	-	-	ppm
Rosin	12.54 ± 0.04	1052150 ± 15080	135189 ± 616	62.40 ± 0.89	ppm

In addition, qualitative screening for other potential marker compounds—herbacetin, tricin, kaempferol, 2-(4-hydroxyphenyl)ethanol (tyrosol), gallic acid, chlorogenic acid, caffeic acid, gossypetin, rhodiocyanoside A, and (2RS)-lotaustralin—also confirmed their absence in the experimental material. Among the identified compounds, only tyrosol was detected as a trace constituent.

### Phase I: effects of repeated testing and stress exposure on exploratory behavior and corticosterone levels (baseline – before stress or treatment)

This first study was conducted to evaluate the effect of mild stress exposure on cortical level and behavioral in the absence of any treatment. The study was conducted on two groups of mice: a stressed group (*n* = 12) and a non-stressed group (*n* = 12), both receiving a placebo. The experimental timeline is reported in Fig. 2.1. During the first week (D1–D6), the mice were acclimated to the caretaker and trained to consume the gummies. On D6, a baseline anxiety level assessment was conducted using behavioral tests (EPM and OF tests). Starting on the eighth day (D8), all animals received a daily dose of the placebo. A second behavioral evaluation was performed on D19, prior to stress induction in order to evaluated to effect of exposition to gummies and daily manipulation. Between D20 and D26, stress was induced to the stressed group, while both groups continued to receive the placebo. On D26, a third and final anxiety level assessment was conducted using the same behavioral tests (EPM and OF tests). Finally, the animals were euthanized, and blood was collected to measure the final corticosterone levels in serum.

**Fig. 2:**
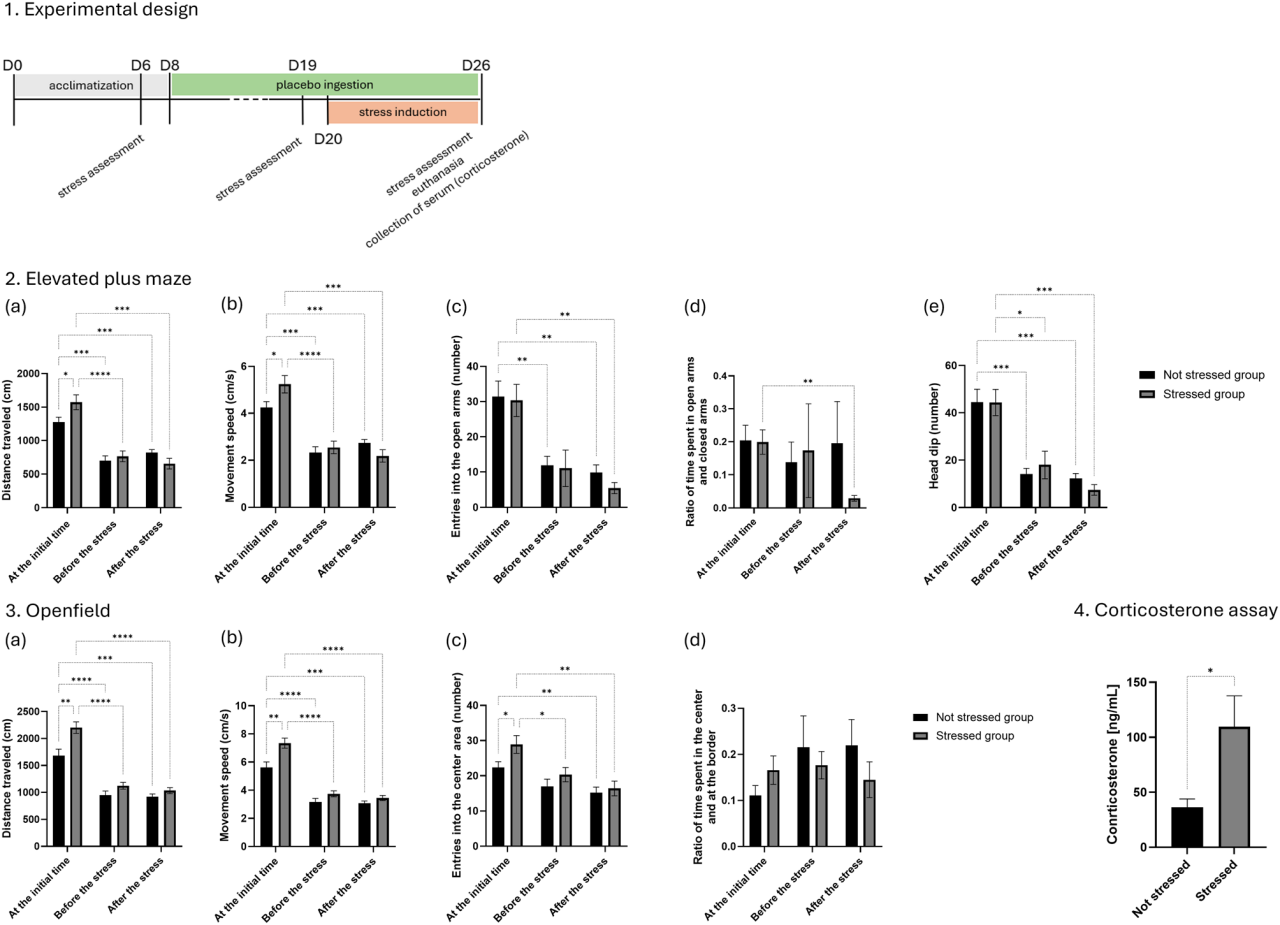
This phase aimed to assess the effects of mild chronic stress on behavior and serum corticosterone levels, in the absence of any treatment. 1. Experimental design of phase I: mice were acclimated for 7 days. A first behavioral stress assessment was performed at day 6 (D6) using the elevated plus maze (EPM) and open Field (of) tests. From day 8 (D8) to day 26 (D26), animals received one placebo gummy per day, administered individually in a separate cage to ensure full ingestion. A second behavioral assessment was conducted at D19, prior to stress induction. From D20 to D26, animals underwent a sequence of mild unpredictable stressors, including : cage tilting, olfactory stress, food and water deprivation, bedding reduction, continuous light exposure, social isolation, and physical restraint. At D26, a final behavioral assessment was followed by sacrifice and blood collection for corticosterone analysis. 2. Elevated plus maze results: (**a**) distance traveled; (**b**) movement speed; (**c**) entries into open arms; (**d**) ratio of time spent in open vs closed arms; (**e**) head dips. 3. Open Field results: (**a**) distance traveled; (**b**) movement speed; (**c**) entries into the center area; (**d**) ratio of time spent in the center vs periphery. 4. Corticosterone assay: serum corticosterone levels at D26. *data are shown as mean ± SEM. *N* = 12 mice per group. Statistical significance: *p < 0.05; **p < 0.01; ***p < 0.001; ***p < 0.0001

The present findings reveal a significant reduction in exploratory behavior in mice as early as the second behavioral assessment, prior to exposure to stress-inducing conditions. These behavioral changes were consistently observed across both the EPM and OF tests, suggesting a robust and early decrease in exploratory activity.

In the EPM test (Fig. 2.2), both stressed and non-stressed groups exhibited a significant reduction in the distance traveled between baseline (D6) and pre-stress (D19). In the stressed group, the distance dropped from 1571.0 ± 110.7 cm to 763.4 ± 80.9 cm, while in the non-stressed group, it decreased from 1276.1 ± 72.9 cm to 697.1 ± 74.1 cm. No significant changes were observed between D19 and post-stress (D26) in either group. A slight initial difference is noted between the stressed (1571.0 ± 110.7 cm) and non-stressed groups (1276.1 ± 72.9 cm). No significant difference is observed between groups either before or after stress exposure.

Movement speed (Fig. 2.2.b) followed the same pattern: a marked decline from D6 to D19 (5.2 ± 0.4 cm/s to 2.5 ± 0.3 cm/s in stressed animals; 4.3 ± 0.2 cm/s to 2.3 ± 0.2 cm/s in non-stressed), with no further change by D26.A slight initial difference is noted between the stressed (5.2 ± 0.4 cm/s) and non-stressed groups (4.3 ± 0.2 cm/s). No significant difference is observed between groups either before or after stress exposure.

The number of entries into open arms (Fig. 2.2.c) also declined significantly between D6 and D19 in both groups (stressed: 30.3 ± 4.6 to 11.1 ± 5.1; non-stressed: 31.4 ± 4.4 to 10.3 ± 2.1), with no significant change at D26. No difference between groups was detected at any point. Regarding the ratio of time spent in open vs. closed arms (Fig. 2.2.d), the stressed group showed a significant reduction only between D6 (0.2 ± 0.0) and D26 (0.0 ± 0.0). The non-stressed group showed no significant variation over time. No significant difference is observed between the stressed and non-stressed groups at the initial time, before, or after stress exposure.

The number of head dips (Fig. 2.2.e) followed a similar trend: a marked decrease from D6 to D19 (stressed: 44.3 ± 5.6 to 18.0 ± 5.8; non-stressed: 44.4 ± 5.6 to 13.3 ± 2.2), with stable values at D26. No significant difference was observed between groups at any time. In the OF test (Fig. 2.3), similar behavioral patterns were observed. Distance traveled (Fig. 2.3.a) decreased significantly between D6 and D19 in both groups (stressed: 2202.2 ± 107.7 cm to 1123.9 ± 62.3 cm; non-stressed: 1684.4 ± 119.1 cm to 943.0 ± 73.8 cm), remaining stable through D26. A slight initial difference is noted between the stressed (2202.2 ± 107.7 cm) and non-stressed groups (1684.4 ± 119.1 cm). No significant difference is observed between groups either before or after stress exposure.

Movement speed (Fig. 2.3.b) decreased significantly from baseline to D19 (stressed: 7.3 ± 0.4 cm/s to 3.7 ± 0.2 cm/s; non-stressed: 5.6 ± 0.4 cm/s to 3.1 ± 0.2 cm/s), but no difference is found between D26 and D19. A slight initial difference is noted between the stressed and non-stressed groups. No significant difference is observed between groups either before or after stress.

The number of entries into the center area (Fig. 2.3.c) declined from D6 to D19 in stressed animals (28.9 ± 2.5 to 20.3 ± 2.0), with no significant change at D26. In non-stressed mice, the decline from D6 (22.4 ± 1.6) to D19 (17.0 ± 2.1) was not statistically significant. A slight initial difference is noted between the stressed and non-stressed groups. No significant difference is observed between groups either before or after stress exposure.

Regarding the center/border time ratio (Fig. 2.3. d), no significant variation was found over time in either group, or between groups at any time point. In contrast to the behavioral findings, corticosterone levels provided clear physiological evidence of stress exposure (Fig. 2.4). At the end of the experiment, the stressed group exhibited an almost threefold increase in circulating corticosterone levels, rising from 36.5 ± 7.5 ng/mL in the non-stressed group to 109.6 ± 28.1 ng/mL (*p* < 0.05). Given that corticosterone is a well-established biomarker of stress in rodents, this substantial elevation confirms the efficacy of the stress induction protocol in eliciting a hormonal stress response.

Exploratory behavior showed a marked decline in mice as early as the second behavioral assessment, even before stress exposure. This reduction was consistently observed across both the Elevated Plus Maze and Open Field tests, affecting distance traveled, movement speed, and open-area exploration. No significant differences emerged between stressed and non-stressed groups at post-stress time points, which could be explained by the mark reduction of mobility observed during the re-test at D9. This suggests that while corticosterone levels nearly doubled in stressed mice, confirming the effectiveness of the stress induction protocol, the repeated behavioral measures were not appropriate. This hypothesis was tested in phase II.

### Phase II: effects of repeated testing on day 14 after stress exposure on exploratory behavior

During the execution of Phase I, we observed significant changes in the animal’s behavior when they were exposed to behavioral tests for the second time. This raised the hypothesis of a potential “test-retest” effect, where prior exposure to the testing environment influences subsequent behaviors. To validate this hypothesis, we conducted Phase II. As shown in the experimental design (Fig. 3.1), the acclimatization period preceded Day 0 (D0), where the first stress assessment was performed on one group of mice (*n* = 14). A second stress assessment took place on Day 14 (D14). Importantly, no interventions occurred between the two tests—mice received no treatment, no candies, and no additional interactions—ensuring that any observed changes were solely attributable to repeated test exposure. These stress assessments were conducted using the Elevated Plus Maze test and the Open Field test. This approach had helped analyze potential changes in behavioral responses over time and had further investigated the “test-retest” effect.

**Fig. 3:**
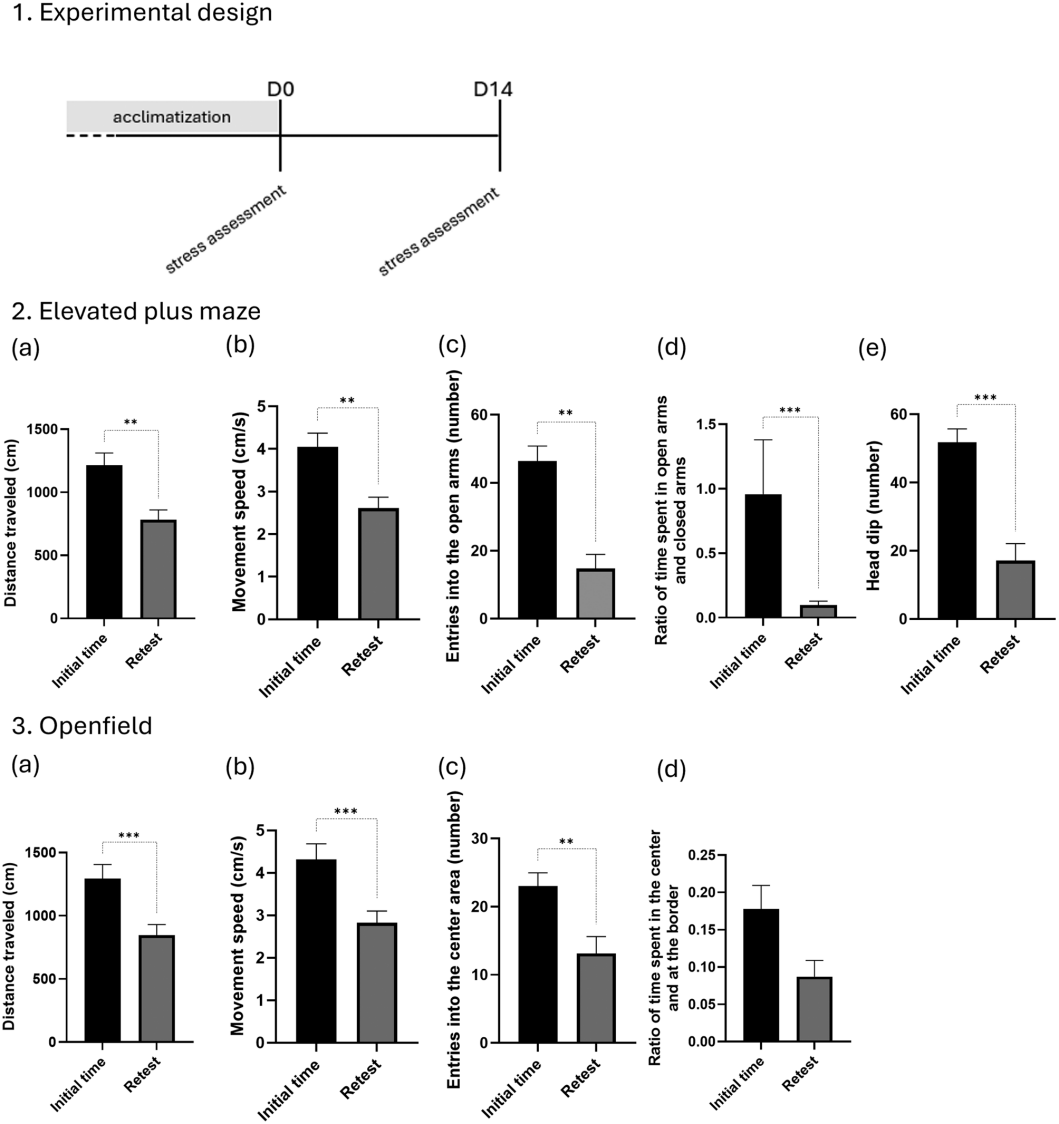
This phase was designed to evaluate the potential habituation of animals to repeated behavioral assessments (elevated plus maze and open Field tests), without stress exposure or treatment. Experimental design: mice were acclimated before the beginning of the experiment. A first behavioral stress assessment was performed on day 0 (D0) using the elevated plus maze (EPM) and open Field (of) tests. No treatment or stress protocol was applied between D0 and D14. A second behavioral evaluation was conducted on day 14 (D14), using the same tests, to investigate the effect of repeated testing and habituation on stress-related behavioral parameters0.2. Exploratory behavior in the elevated plus maze test: (**a**) distance traveled; (**b**) movement speed; (**c**) entries into the open arms; (**d**) ration of time spent in open arms and closed arms; (**e**)head dip. 3. Exploratory behavior in the Openfield test: (**a**) distance traveled; (**b**) movement speed; (**c**) entries into the center area; (**d**) ratio of time spent in the center and at the border. Data are shown as mean ± SEM. *N* = 14 mice per group. Statistical significance: **p < 0.01; ***p < 0.001

The results reveal a significant decrease in exploratory behavior in mice between the initial test and the retest, as assessed in both the Elevated Plus Maze (EPM) and Open Field (OF) tests.

In more details (Fig. 3.2), in the EPM, locomotor activity declined markedly, as shown by a significant reduction in total distance traveled from 1215.4 ± 97.0 cm to 783.7 ± 77.4 cm, reflecting decreased exploratory drive upon repeated exposure. Similarly, the movement speed (Fig. 3.2.b) decreasing from 4.1 ± 0.3 cm/to 2.6 ± 0.3 cm/s. The number of entries into the open arms (Fig. 3.2.c) was markedly reduced, from 46.4 ± 4.5 to 14.8 ± 4.2, reinforcing the habituation effect to the testing environment. Similarly, the ratio of time spent in open versus closed arms (Fig. 3.2.d) showed a pronounced reduction, dropping from 1.0 ± 0.4 to 0.1 ± 0.0, suggesting an increased preference for enclosed areas over open, anxiogenic spaces. Risk-taking behaviors, such as head dips (Fig. 3.2.e), also significantly declined from 51.8 ± 3.9 to 17.1 ± 5.0, supporting the overall reduction in exploratory motivation.

Comparable results were observed in the OF test (Fig. 3.3). Total distance traveled decreased from 1294.5 ± 111.5 cm to 847.0 ± 84.1 cm, and movement speed similarly dropped from 4.3 ± 0.4 cm/s to 2.8 ± 0.3 cm/s, reinforcing the habituation effect. The number of entries into the center zone (Fig. 3.3.c) significantly decreased from 23.0 ± 2.0 to 13.1 ± 2.5, indicating a lower tendency to explore central, anxiogenic areas. The ratio of time spent in the center versus the periphery (Fig. 3.3.d) showed a slight non-significant decline, suggesting an increased preference for remaining near the periphery rather than venturing into the central area. This behavioral shift likely reflects a reliance on previously explored zones as the mice adapted to the environment.

The results of phase II demonstrate a pronounced habituation effect, characterized by a reduction in exploratory behavior during the retest.

*Phase III: impact of Rhodiola rosea L. roots powder on optimized murine model (Optimization after 14 days of stress exposure with treatment using Rhodiola rosea L. root powder (administered from day 15 to day 33)*

In final phase (Phase III), our primary objective was to evaluate whether *Rhodiola rosea L.* treatment could mitigate the behavioral and physiological consequences of chronic stress. For this reason, the experimental design was focused exclusively on stressed animals, as they represent the most relevant condition for testing adaptogenic and anxiolytic effects. Including a non-stressed group with or without treatment would have provided additional information regarding baseline effects of *Rhodiola rosea L.*; however, due to ethical considerations and in strict compliance with European Directive 2010/63/EU on the protection of animals used for scientific purposes, as well as its transposition into Belgian law [62], our study design followed the 3Rs principle (Replacement, Reduction, Refinement) [63]. Specifically, the exclusion of additional non-stressed groups was based on the reduction principle, aiming to limit the number of animals used while still achieving scientifically valid results (Phase III was restricted to stressed groups only).

The final phase of the study was conducted on two groups of 12 mice: one group treated with *Rhodiola rosea L.* (800 mg/kg/day) and a control group treated with placebo. The first 14 days (D1 to D14) were dedicated to acclimation, including handling and habituation to the gummies. From Day 15 (D15) onward, the mice received either *Rhodiola rosea L.* (treated group) or a placebo (control group), and this administration continued until Day 33 (D33). All mice were exposed to the stress protocol between Days 27 (D27) and 33 (D33), while mice continued receiving their respective treatments. To prevent the test-retest effect, which was observed during Phase II, a single behavioral assessment was conducted at D33 using the Elevated Plus Maze (EPM) and the Open Field (OF) tests to evaluate anxiety-related behavior. Finally, the mice were euthanized, and serum corticosterone levels were measured to correlate behavioral observations with physiological stress responses.

The results suggest an overall increase in exploratory activity and a reduction in behavioral inhibition following *Rhodiola rosea L.* treatment.

In more details, in the EPM (Fig. 4.2), locomotor activity was greater in the treated mice, as reflected by an increase in the total distance traveled (Fig. 4.2.a) from 921.0 ± 78.3 cm (placebo) to 1416.2 ± 86.2 cm (treated), along with an increase in movement speed (Fig. 4.2.b) from 3.1 ± 0.3 cm/s to 4.7 ± 0.3 cm/s. Treated animals also made more entries into the open arms, increasing from 11.3 ± 2.9 to 36.9 ± 4.3, and spent more time in open versus closed arms, with a ratio rising from 0.1 ± 0.0 to 0.5 ± 0.0, indicating reduced avoidance of anxiogenic areas. and suggesting reduced anxiety-like behavior. In addition, risk-taking behaviors such as head dips were significantly more frequent in treated mice, nearly doubling from 13.3 ± 3.0 to 43.2 ± 4.4, suggesting enhanced proactive exploration.

**Fig. 4:**
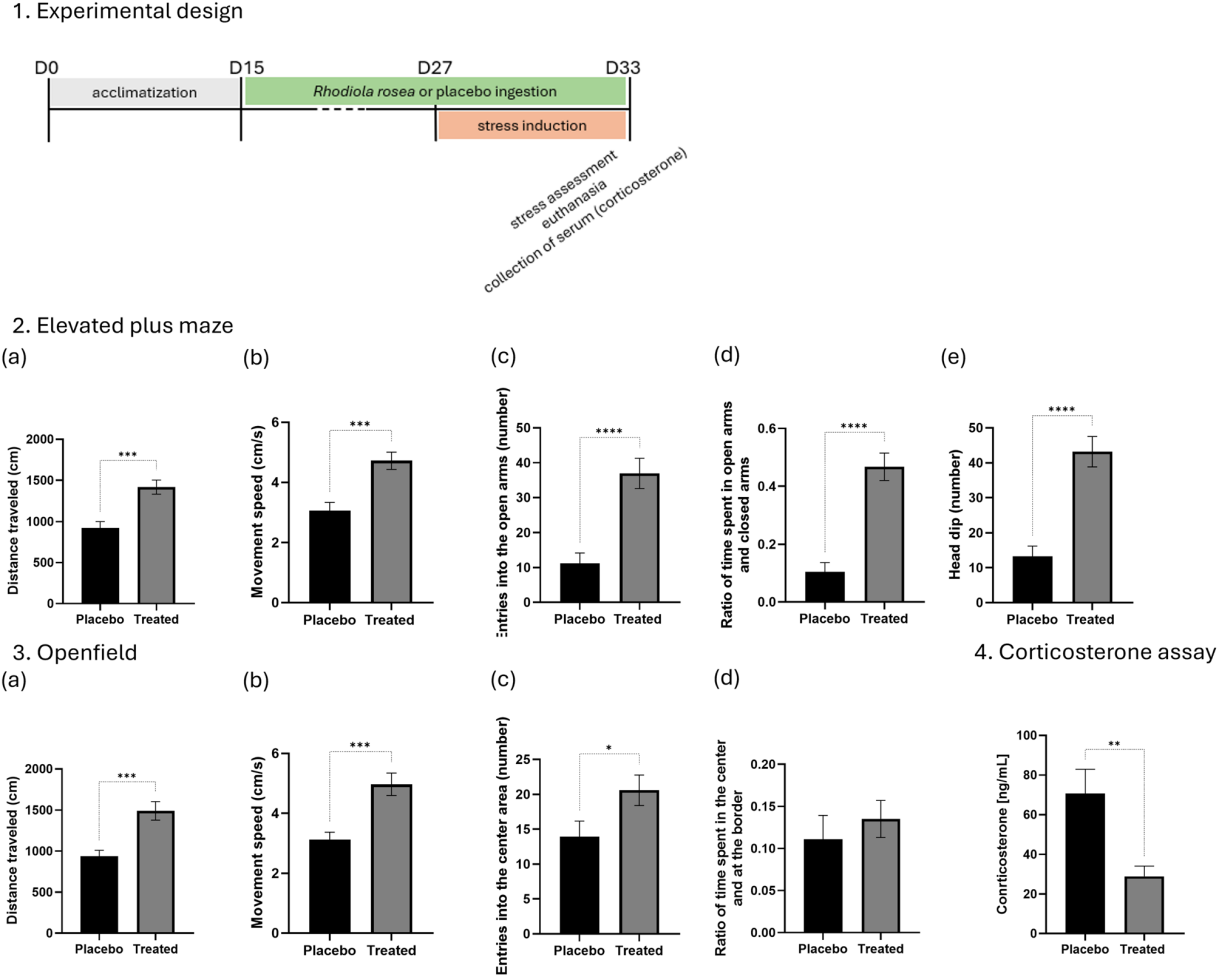
This final phase compared a treated group and a placebo group, both subjected to the same chronic mild stress protocol, as well as a non-treated, non-stressed control group. The aim was to assess the efficacy of Rhodiola rosea root powder in modulating behavioral and physiological stress responses in comparison to untreated animals. 1. Experimental design: mice were first acclimated for 14 days (D0–D14). From Day 15 (D15) to Day 33 (D33), animals received one gummy per day containing either Rhodiola rosea root powder (enriched with 3% salidroside) or a placebo. Gummies were administered individually to each mouse in a separate cage to ensure complete ingestion. From D27 to D33, animals were subjected to a series of mild, variable stressors: cage tilting, olfactory stress, food and water deprivation, bedding reduction, continuous light exposure, social isolation, and physical restraint. On D33, behavioral testing was performed (elevated plus maze and open Field tests), followed by euthanasia and blood collection for serum corticosterone analysis. 2. Exploratory behavior in the elevated plus maze test: (**a**) distance traveled; (**b**) movement speed; (**c**) entries into the open arms; (**d**) ration of time spent in open arms and closed arms; (**e**)head dip. 3. Exploratory behavior in the Openfield test: (**a**) distance traveled; (**b**) movement speed; (**c**) entries into the center area; (**d**) ratio of time spent in the center and at the border. 4. Corticosterone assay. Data are shown as mean ± SEM. *N* = 12 mice per group. Statistical significance: *p < 0.05; **p < 0.01; ***p < 0.001; ***p < 0.0001

In the OF test, the beneficial effects of *Rhodiola rosea L.* treatment were also evident (Fig. 4.3). The locomotor activity was enhanced in the treated mice, with total distance traveled (Fig. 4.3.a) increasing from 938.2 ± 72.4 cm (placebo) to 1490.3 ± 111.8 cm (treated), and movement speed (Fig. 4.3.b) rising from 3.1 ± 0.2 cm/s to 5.0 ± 0.4 cm/s. Treated animals also showed a higher number of entries into the center zone (13.9 ± 2.3 vs. 20.6 ± 2.2), indicating reduced avoidance of anxiogenic central areas. Although the ratio of time spent in the center versus the periphery (Fig. 4.3.d) did not differ significantly between groups, a trend toward a decrease in this ratio was observed in the untreated group, suggesting a preference for the periphery in untreated mice.

Finally, corticosterone levels (Fig. 4.4) confirmed a physiological reduction in stress response, with treated mice showing significantly lower concentrations (28.9 ± 5.2 ng/mL) compared to placebo-treated animals (70.6 ± 12.3 ng/mL; *p* < 0.01), consistent with the observed behavioral improvements.

**Fig. 5:**
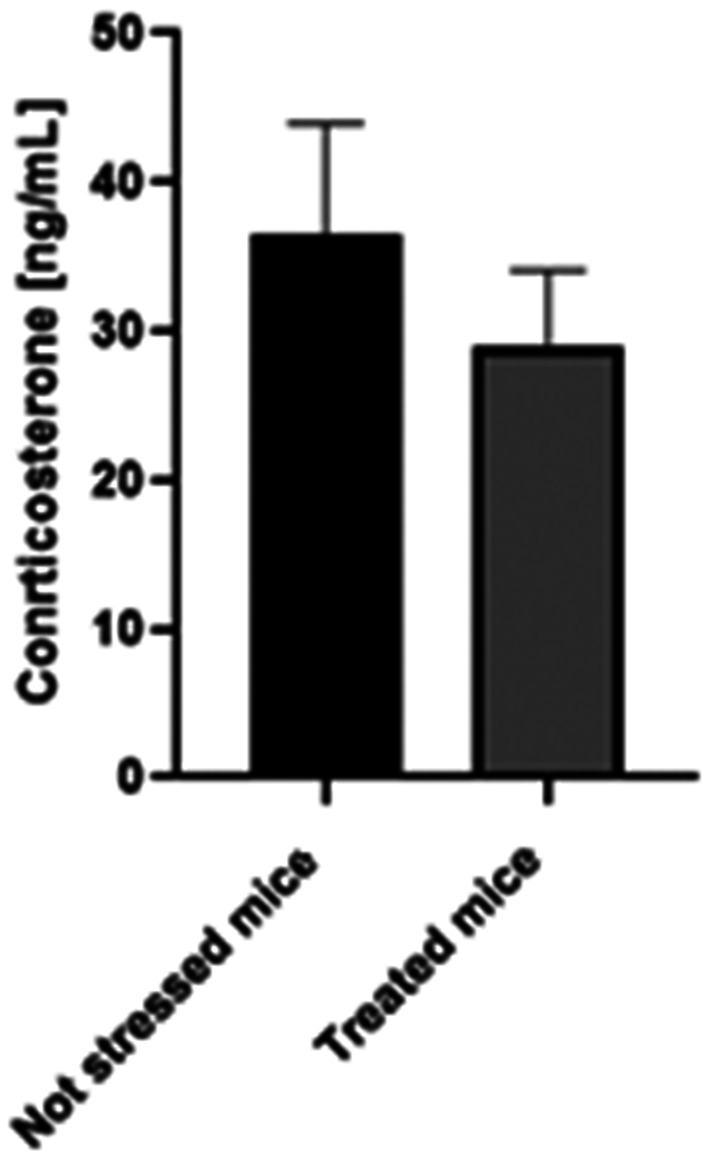
Comparison of corticosterone levels in non-stressed mice treated with placebo and stressed mice treated with *Rhodiola rosea L. N* = 12

Comparison of these corticosterone levels with those obtained in Phase I suggests that stressed mice treated with *Rhodiola rosea L.* (28.9 ± 5.2 ng/mL) exhibited corticosterone levels like those of non-stressed mice (36.5 ± 7.5 ng/mL) (Fig. 5). These findings reinforce the hypothesis that *Rhodiola rosea L.* exerts an adaptogenic effect by mitigating both behavioral and physiological responses to stress.

## Discussion

The present study aimed to evaluate the effects of *Rhodiola rosea L.* root powder on stress-related behavioral and physiological responses in mice. While most existing research has focused on *Rhodiola rosea L.* extracts, particularly standardized formulations containing 3% rosavin and 1% salidroside [40], this study investigated the impact of a *Rhodiola rosea* L. root powder formulation with 3% salidroside. This approach allowed for a direct assessment of the adaptogenic properties of the plant in its powdered form, while also providing new insights into its efficacy in mitigating stress-induced alterations in behavior and corticosterone levels. The adaptogenic properties of *Rhodiola rosea*, defined as its capacity to enhance the organism’s resistance to stress, are widely regarded as the result of a complex interaction among multiple phytochemical constituents rather than the action of a single active compound. The presence of numerous constituents, including those occurring only in trace amounts, may play a critical role in shaping the overall biological activity of the plant. Variations in cultivation, environmental conditions, and processing can alter the phytochemical profile, thereby influencing the physiological effects observed in vivo [64].

In the present study, *R. rosea* root powder was produced using an indoor cultivation system that ensures tightly controlled and reproducible growth conditions. This approach minimizes variability and enables a consistent phytochemical fingerprint. The multifactorial nature of *R. rosea* bioactivity also underlies current quality-control practices, which typically rely on both rosavins and salidroside—phenylpropanoid and phenylethanoid derivatives—as key marker compounds [65]. The formulation examined here, however, consists of whole root powder standardized to 3% salidroside, representing a distinct composition compared with conventional market extracts that are usually enriched in rosavins.

The unique phytochemical complexity of the root powder, including its trace constituents, may contribute to the biological effects observed and supports the hypothesis of synergistic interactions among components [66]. These findings establish a basis for future mechanistic studies aimed at isolating individual molecules and formally characterizing synergistic or additive interactions. Overall, the results demonstrate that *Rhodiola rosea* L. root powder (with 3% salidroside) exerts a significant modulatory effect on both physiological and behavioral markers of stress.

The results from the first phase of the study highlighted a reduction in exploratory behavior in both stressed and non-stressed mice, even before exposure to the stress. This decline underscores the importance of considering habituation effects when interpreting behavioral outcomes, as repeated exposure to the same test environment can lead to decreased exploratory activity independent of stress induction. The second phase further confirmed this habituation effect. These results emphasize the complexity of interpreting behavioral changes, as habituation can obscure the direct impact of stress, highlighting the necessity of accounting for this effect in stress-related studies and integrating both physiological and behavioral measures for a more comprehensive analysis. The study by Almeida et al. clearly shows that repeated exposure to the Elevated Plus Maze leads to a significant reduction in both the number of entries and the time spent in the open arms. This finding suggests that increasing familiarity with the test environment can dampen exploratory behavior. These results are in line with those reported by Lee and Rodgers and Rodgers et al. who also observed decreased open-arm exploration upon reexposure [67–69]. In contrast, earlier studies by Pellow et al. [70] and Chappell et al. [71] did not find any notable changes in these parameters across repeated sessions, highlighting possible methodological differences or variations in experimental design [70, 71].

In the final phase, the effects of *Rhodiola rosea L.* root powder on stress-induced behavioral and physiological changes were assessed. Mice receiving the treatment displayed enhanced exploratory activity, increased open-arm exploration in the Elevated Plus Maze, and greater center exploration in the Open Field test compared to placebo-treated mice. These behavioral changes were accompanied by a significant reduction in corticosterone levels, indicating a diminished physiological stress response. Notably, corticosterone concentrations in treated mice were comparable to those observed in non-stressed animals from Phase I, further supporting the adaptogenic potential of *Rhodiola rosea L.*.

While treated mice showed increased locomotor activity—evidenced by higher movement speed and greater distance traveled—this could, in theory, be attributed to a stimulant-like effect of the plant rather than a true anxiolytic response. Elevated motor activity alone does not necessarily imply reduced anxiety, as an animal may remain anxious despite being more active. However, anxiety-related behaviors are more accurately assessed using specific indicators such as the ratio of time spent in the center versus the periphery in the Open Field test, the ratio of time spent in open versus closed arms in the Elevated Plus Maze, and the number of entries into these areas. In this study, these anxiety-related measures were closely linked to locomotor activity data. Since treated mice not only moved more but also entered open or central areas more frequently, the results strongly support the idea that *Rhodiola rosea L.* produces an adaptogenic effect rather than a mere excitatory response and further reinforce its potential as a natural modulator of stress. Moreover, the movement speeds recorded during the first test of Phase I and Phase II, as well as the speed observed in the treated group during Phase III, remained consistent across both the Open Field and Elevated Plus Maze tests. This stability in locomotor activity further reinforces the conclusion that the plant’s effect is adaptogenic in nature, rather than simply stimulating, strengthening the overall interpretation of its stress-modulating properties.

Furthermore, movement speeds recorded during the first tests of Phase I and Phase II, as well as the speed observed in the treated group during Phase III, remained stable across both behavioral paradigms. This consistency in locomotor activity further supports the interpretation that the plant’s effects are adaptogenic rather than simply stimulating.

Nevertheless, the study has a some limitation. The experimental design did not include a dedicated group of non-stressed animals treated with *Rhodiola rosea* L. to evaluate the plant’s effects in the absence of stress. Such a group would have been essential to fully exclude the possibility of subtle psychostimulant effects and to better isolate the treatment’s intrinsic behavioral impact, particularly on locomotion. It was provided a reasonable justification for this omission, acknowledging it highlights an important avenue for future research. Future studies should therefore include a non-stressed, *Rhodiola*-treated group to directly assess the baseline behavioral influence of the extract and to further clarify its adaptogenic versus stimulant properties.

The results of this study demonstrate that *Rhodiola rosea* L. root powder significantly influences behavioral and physiological stress responses in mice. These findings align with prior research showing that *Rhodiola rosea* L. regulates stress-related gene expression, reduces corticosterone levels, and mitigates stress-induced disruptions in the brain and immune system [43, 47, 72]. Similar adaptogenic effects were also reported by Shikov et al. [73], who observed increased physical endurance and a reduction in anxiety-associated behaviors such as grooming following a 7-day oral administration of a liquid *Rhodiola rosea* L. extract. However, in their study, anxiolytic effects in the light/dark and open-field tests did not reach statistical significance. This discrepancy may be attributed to several methodological differences, including the treatment duration, the type and dosage of *Rhodiola rosea* L. administered, and the testing conditions. These factors likely contributed to the more robust anxiolytic and physiological effects observed in our chronic stress model, notably the significant increases in exploratory behaviors and the marked reduction in corticosterone levels.

*Rhodiola rosea* L. is recognized as an adaptogen that enhances stress resilience. Studies have reported its anxiolytic and antidepressant effects, with evidence showing improved behavioral responses and reduced corticosterone levels following chronic mild stress [34, 45, 74, 75]. Its active compound, salidroside, counteracts inflammation through inhibition of the P2X7/NF-κB/NLRP3 pathway [76], helping to restore homeostasis disrupted by chronic stress [77–79]. *Rhodiola rosea* L, recognized for its adaptogenic properties, modulates corticosterone production by influencing the hypothalamic–pituitary–adrenal (HPA) axis during periods of stress. Evidence suggests that *Rhodiola rosea* L. extract can attenuate the hyperactivity of the HPA system, thereby regulating corticosterone release. Under stress conditions, the hypothalamus secretes corticotropin-releasing hormone (CRH), which stimulates the anterior pituitary to release adrenocorticotropic hormone (ACTH), ultimately triggering the adrenal glands to secrete corticosterone [80–83]. This pathway is mediated through glucocorticoid receptors, which modulate stress-responsive gene expression [84].

The adaptogenic potential of *Rhodiola rosea* L. depends on its dosage and composition [85]. In this context, our study demonstrated that a newly developed *Rhodiola rosea* L. root powder, with a high concentration of salidroside (3%), effectively mitigates stress-induced behaviors. To further explore the potential of *Rhodiola rosea* L., future research could focus on comparing the effects of root powder with those of standardized extracts. While our results confirm the efficacy of the root powder formulation, investigating whether its effects differ from commercial extracts would provide valuable insights into its specific adaptogenic properties. If both formulations yield comparable results, the choice of root powder may offer additional advantages. Unlike extracts, which require the use of solvents for the extraction process, root powder maintains the plant’s natural composition without the need for chemical processing. This aligns with the current trend toward greener, more sustainable solutions in natural health products, reducing the environmental impact associated with solvent use while preserving the full spectrum of bioactive compounds naturally present in the plant.

The concept of hormesis, defined as a biphasic response to a bioactive substance with stimulatory effects at low doses and inhibitory effects at high doses, has recently been discussed in the context of the biological activity of *Rhodiola rosea* L. Several in vitro studies conducted on unicellular models (such as *Saccharomyces cerevisiae*) have highlighted hormetic responses to *Rhodiola rosea* L. extract or its major active compound, salidroside [86–88]. These studies show beneficial effects at low doses on longevity, oxidative stress resistance, or cell survival, while higher doses induce opposite or even deleterious effects. Nevertheless, most of this research has been conducted in vitro or on highly simplified models, and few in vivo studies have directly assessed the existence of a hormetic effect in the context of chronic stress. Moreover, the available studies focus more on longevity or cellular protection than on behavioral or neuroendocrine effects related to stress. In this work, although we did not systematically explore a range of doses to characterize a potential hormetic response, our results indicate that a high and prolonged dose of *Rhodiola rosea* L. root powder enriched in salidroside (3%) produces significant anxiolytic and anti-stress effects. It would be relevant in future studies to test different doses and treatment durations in order to determine whether a biphasic dose–response relationship also appears in behavioral models of chronic stress.

This study provides promising evidence for the adaptogenic properties of *Rhodiola rosea* L., yet several limitations should be acknowledged to accurately interpret the findings. Biologically, the investigation focused solely on corticosterone levels, without a broader assessment of the hypothalamic-pituitary-adrenal (HPA) axis, limiting insight into the precise site of action. Behaviorally, the study did not address additional domains such as fine motor function or cognition, which could further contextualize the observed effects. Moreover, the fixed treatment duration and single high-dose regimen preclude conclusions about long-term efficacy or dose–response relationships. These limitations underscore the need for further targeted studies to refine our understanding of *Rhodiola rosea* L.’s adaptogenic potential.

Overall, the observed reductions in stress-related behaviors and corticosterone levels suggest that *Rhodiola rosea L.* root powder may help mitigate the effects of chronic stress and enhance adaptation to stress-inducing conditions. These findings contribute to the broader field of research on plant-derived adaptogens and highlight *Rhodiola rosea L.* as a promising natural intervention for stress-related disorders. Further investigations should examine the long-term effects of *Rhodiola rosea L.* supplementation, its influence on additional physiological markers of stress, and its mechanisms of action at the molecular level.

## Data Availability

The datasets used and/or analysed during the current study are available from the corresponding author on reasonable request.
